# Relationships among Healthcare Digitalization, Social Capital, and Supply Chain Performance in the Healthcare Manufacturing Industry

**DOI:** 10.3390/ijerph18041417

**Published:** 2021-02-03

**Authors:** Hee Kyung Kim, Chang Won Lee

**Affiliations:** 1 GTB Center, School of Business, Hanyang University, Seoul 04763, Korea; kimhk1866@hanyang.ac.kr; 2 School of Business and Healthcare MBA Program, Hanyang University, Seoul 04763, Korea

**Keywords:** healthcare digitalization, healthcare manufacturing, social capital, supply chain performance

## Abstract

Due to the impact of coronavirus disease 2019 (COVID-19), automation and artificial intelligence (AI) have attracted renewed interest in multiple industrial fields. Global manufacturing bases were affected strongly by workforce shortages associated with the spread of COVID-19, and are working to increase productivity by embracing digital manufacturing technologies that take advantage of artificial intelligence and the Internet of Things (IoT) that offer the promise of improved connectivity among supply chains. This trend can increase and smooth the flow of social capital, which is a potential resource in supply chains and can affect supply chain performance in healthcare industry. However, such an issue has not been properly recognized as the best practice in healthcare industry. Thus, this study investigates empirically the relationship between digitalization and supply chain performance in healthcare manufacturing companies based on previous research that proposed a role for social capital. We surveyed the staff of domestic small and medium-sized healthcare manufacturing companies in South Korea currently operating or planning to deploy digital manufacturing technologies. Online and email surveys were utilized to collect the data. Invalid responses were excluded and the remaining 130 responses were analyzed using a structural equation model in SPSS with the AMOS module. We found that digitalization has a positive effect on the formation of social capital, which in turn has a positive effect on supply chain performance. The direct effect of digitalization on supply chain performance is small, and relatively large portions are mediated and influenced by social capital. The establishment of strategic relationships in the healthcare manufacturing industry is significant, as supply chain networks and production processes can influence the intended use of factory output. Companies should, therefore, secure timely and accurate information to manage the flow of products and services. The formation of social capital in the supply chain can help visualize entire supply chains and has a positive effect on real-time information-sharing among key elements of those chains.

## 1. Introduction

Personal lives and entire industrial ecosystems are under pressure from the effects of coronavirus disease 2019 (COVID-19). Experts in healthcare, transportation, logistics, and manufacturing industries are anticipating that the use of automation and artificial intelligence (AI) will continue to spread in response to the pandemic’s effects on human workforces [1]. In the manufacturing industry, in particular, the globalization of existing production bases has been disrupted by COVID-19 and value chains are strengthening around region or countries where companies are located. According to United Nations Conference on Trade and Development (UNCTAD), strengthening regional value chains should be a priority for developing countries to diversify risk, reduce vulnerability, increase resilience and foster industrial development. By identifying and maintaining horizontal and vertical linkages, regional pacts can ensure that small firms cooperate to reduce transaction costs and benefit from economies of scale. As manufacturing costs continue to increase, and production workforces shrink, companies are hoping to increase productivity by incorporating information and communications technology into their operations [2,3]. As a result, big data collected through the Internet of Things (IoT) and technology that simulates real-world conditions and predicts the results are attracting attention [4]. In healthcare manufacturing, technology and medical trends affect each other as well as the production and supply processes, which benefit from simple and convenient workflows that can be facilitated by digital manufacturing technologies [5,6,7].

Digitalization of supply chains in the healthcare manufacturing industry has improved connectivity, and the flow of information and potential resources through the network has been smoothed [8,9]. A supply chain is defined as a series of interdependent relationships among suppliers and customers in upstream side and downstream side in supply chain developed through strategic coordination and responsiveness. The potential resources arising from supply chain flow can be explained in terms of social capital [10,11]. Previous studies suggest that improvement in supply chain performance depends on whether social capital is implied from a supply chain perspective. In addition, it has been argued that the density of a supply chain increases according to the degree of social capital integration. Because social capital has similarities to routine knowledge-sharing, the performance of a supply chain can be enhanced through regular and intimate interactions between suppliers and consumers [12,13,14,15].

Based on the role of social capital in supply chains described by previous studies, the purpose of this study is to explore the effects of digitalization on supply chain performance in terms of the accumulation and flow of social capital. Healthcare manufacturing, in which products and purpose of use are strongly influenced by trends in technologies, is selected as the primary focus of this study. In addition, we explore the prospects for strengthening domestic companies’ supply chain management (SCM) expertise through the application of digital manufacturing technologies and how connections among industries can be improved.

This study comprises six sections. Section 1 presents study motivation and the study purpose. Section 2 reviews various literatures related to this study and develops pertinent hypotheses along with a study model. Section 3 describes sampling procedures and statistical methods to conduct empirical tests. Section 4 presents the results of analysis of significant impacts using a structural equation model (SEM) to specify the relationship between digitalization and supply chain performance, and explore the mediating role of social capital. Section 5 critically examine and explores the main findings. Section 6 presents a summary of this study and implications for the role of social capital and the impact of digitalization on healthcare manufacturers are suggested, along with practical recommendations for further research.

## 2. Literature Review and Hypotheses Development

### 2.1. Healthcare Digitalization

This study aims to examine the impact of digitalization on healthcare manufacturing. Digitalization is the use of information technologies to facilitate intra- and inter-organizational decision-making, processes, and architectures [16]. Prior to healthcare digitalization, theories related to digitization and other processes associated with the conversion of analog information management to digital platforms had attracted research attention [17]. However, as AI, augmented reality (AR), and the IoT have been applied to manufacturing processes, a shift from healthcare digitization to digitalization began to take place. Applying a digitization theory to digitalization is therefore no longer appropriate [18]. The digitalization of intra- and inter-organizational processes offers significant opportunities for research of SCM [19].

Healthcare digitalization can be separated into three dimensions: internal efficiency, disruptive change, and external opportunities. Healthcare digitalization scale items were based on a study of systematic change of organizations by Parviainen et al. [20], who shared the theoretical underpinning of this study. Healthcare digitalization was divided into three factors: internal efficiency, disruptive change, and external opportunities. Internal efficiency was measured by accessibility to markets and customer information, strategic plan feasibility, expected cost-reduction effects, employee competence, and technology development. Disruptive change was gauges by whether a firm works with existing or new business partners, and by business flexibility. External opportunities were measured by the degree of communication with internal and external stakeholders, capturing the opportunities of new market and business.

Internal efficiency can be defined as an improved way of using digital means and re-planning internal processes, one that can affect a company’s entire operating environment and internal functioning. This includes improving business-process efficiency, quality, and consistency by eliminating manual steps and refining accuracy [21,22]. Digitalization can also improve real-time views of operations and results by integrating structured and unstructured data, and data from other sources [23].

Disruptive change can be defined as complete change. Disruptive change in healthcare digitalization involves the operators in a value chain, and culminates in an existing business. Traditional intermediates in a supply chain can be removed or replaced by new intermediates [24]. Automation of routine tasks can improve worker satisfaction as time is freed up to develop new skills. Disruptive changes in healthcare digitalization can improve compliance through standardized record-keeping, easier data backups, and distributed storage [25,26].

External opportunities can be defined as new business opportunities in existing domains. External opportunities in healthcare digitalization include improved response times and client services, as well as new ways of doing business [27]. New digital manufacturing technologies can create opportunities for new services or advanced offerings to customers. Opportunities created by the healthcare digitalization are examined along with the resulting implications for inventory management and after-sales operations [28,29]. Applying digital manufacturing technology to healthcare industry makes a possible feedback-loop autonomous state in healthcare related businesses. It implies a sustainable model related to self-regeneration, recycling and movement of resources. Therefore, the use of digital manufacturing technology should aim not only at improving business processes, but also their sustainability. Building continuous improvement by digitalization can ensure that a satisfactory innovation framework supports business needs, builds a system of overseeing innovation assets, and opens doors to innovation made possible by potential changes and impacts [30]. Digitalization allows for the distribution of assets that can organize, improve, and deliver resources and management. The ability to implement digitalization can improve performance access to markets and consumers [31,32].

### 2.2. Social Capital and Supply Chain Performance

The concept of social capital was introduced by economists in the 1990s. Social capital theory directs attention toward the potential of a firm’s social networks to provide a competitive advantage [33]. Formal social capital has been shown to generate more benefits in the form of financial resources when compared with informal social capital [34]. Training in production, operations, and planning, together with formal social capital, was reportedly common among entrepreneurs exploiting high-growth resources [35]. The impact of social capital on supply chain performance has been studied at multiple levels using different performance measures.

Social capital is widely described in the literature as a valuable asset that stems from access to resources made available through social relationships. Three dimensions of social capital: relational, cognitive, and structural. They proposed that relational capital concerns relationships among people with a history of interactions [36]. This capital, which encompasses the character and qualities of the connections between individuals, is often characterized by trust, cooperation, and the identity that a particular individual has within a network of relationships [37,38]. The interactions can be influenced by the relationship and history of exchanges between particular individuals. Cognitive capital refers to resources that provide shared representations, interpretations, and systems of meaning among parties. This capital captures the concepts of shared norms, systems of meanings and values, and, as such, can be expected to directly affect the development of both social capital and relationships [39,40]. Structural capital includes the properties of a social system and the network of relationships. It includes network components and facets such as the presence or absence of ties between parties; the configuration of a network, such as the hierarchy within an organization; concepts such as the density of relationships, structural holes in networks, the presence or absence of network ties among different people and formal and/or (appropriable) informal network configurations; and the density and connectivity of a network [41,42].

The role of social capital in buyer-supplier relationships and its impact on supply chain performance has been studied from the SCM perspective [43]. This study also presumes that social capital has considerable influence on supply chain performance. Previous studies have indicated that social capital plays a central role in driving performance improvement in supply chains. Social capital enables superior planning and the setting of specific goals and problem solving, and can therefore enhance supply chain performance. Interaction between suppliers through technical exchanges can also help improve supply chain performance. In short, social capital encouraged by information-sharing, communication, and joint problem-solving exercises, along with trust, partnership, and familiarity, can provide supply chain members with opportunities to improve performance [44].

Social capital was established as a mediated variable based on studies by Krause et al. [45] and Roden and Lawson [39] in which social capital was divided relational, cognitive, and structural dimensions. Relational capital was measured by the degree of closeness of the interactions, mutual trust, mutual friendship, and reciprocity at multiple levels. Cognitive capital was measured in terms of whether the respondents shared the business values, ambitions, and vision and understood the company’s goals for the business. Structural capital described the items related to relationship characterized by social events. Metrics included joint execution and the holding of workshops, cross-functional teams, co-location, and team-building activities with partnership firms.

On healthcare digitalization, social capital is the fundamental asset involved in designing, creating, and offering assistance and items to meet business needs [46]. Social capital consists of a system structure and potential assets that can pass through the system. It is both an asset and a resource that constitutes a relationship in a supply chain. Social capital among organizations leads to self-development. Established relationship patterns and communication processes benefit from technological facilities and infrastructure that create dynamic opportunities. Because social capital provides external sources of information and knowledge, it has a significant effect on value creation and affects healthcare digitalization and performance [47,48].

Supply chain performance was based on studies by Singhal et al. [49] and Terjesen et al. [50]. Supply chain performance can be affected by the operational perspective of supply chain processes and bullwhip effect minimization, including agility (production flexibility and order-delivery cycle time reduction), capacity (the driving force to explore new products and new markets), and product diversity (the degree of provision of various products according to market demand and globalization).

**Figure 1:**
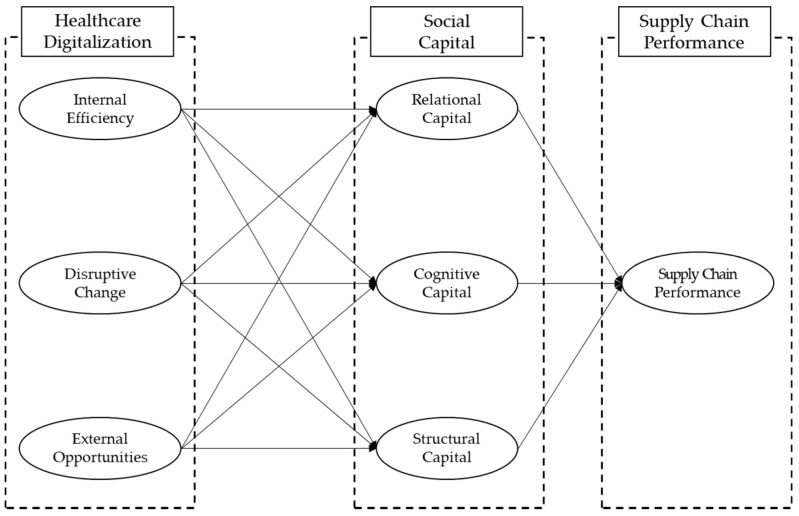
The study model.

Based on the above review, a relationship identifies among healthcare digitalization and social capital, as well as social capital and supply chain performance. These relationships are drawn in Figure 1 and are summarized in the following hypothesis:

**Hypothesis** **1** (**H1**)**:** Healthcare digitalization (internal efficiency, disruptive change, external opportunities) is positively associated with Social capital (relational, cognitive, structural).

**Hypothesis** **2** (**H2**)**:** Social capital (relational capital, cognitive capital, structural capital) is positively associated with supply chain management.

**Hypothesis** **3** (**H3**)**:** The relationship between healthcare digitalization (internal efficiency, disruptive change, external opportunities) and supply chain performance is mediated by Social capital (relational capital, cognitive capital, structural capital).

## 3. Methodology

To test the research model, we collected data from 130 respondents working at small and medium-sized healthcare manufacturing companies, especially medical equipment, in South Korea. Online form and emails were utilized to collect the data. The survey period, including pre-test surveys, ran from 5 September 2020, to 24 October 2020. A total of 152 responses were collected. Invalid responses were excluded and the remaining 130 were analyzed. We performed statistical analyses using SPSS 21.0 with the AMOS 22.0 module.

Common method bias (CMB) and non-response bias are important factors that can distort correlations between independent and dependent variables, and undermine internal validity. Because this study collected data from a self-administered online survey, CMB was possible. To minimize the chances of this occurring, an explanation on the concept of measurement variables was included in the survey, and the questionnaire was clarified through pre-tests. In addition, non-response data that were difficult to resolve completely were excluded.

Descriptive statistics analysis and frequency analysis were conducted. In addition, confirmatory factor analysis and reliability analysis (using Cronbach’s alpha) were used to verify variable validity, and discriminant validity was determined using average variance extracted and construct reliability values. To confirm correlations between factors in the variables, we calculated Pearson’s correlation coefficients. Finally, to verify the hypotheses, determine model fit, and assess multicollinearity problems, a Structural Equation Modeling (SEM) analysis was implemented. The analysis variables were revised and supplemented with existing empirical and case studies and verified by comparisons with previous studies. All variables, with the exception of demographic items, were measured using a five-point Likert scale and provided in Appendix A. 

## 4. Results

### 4.1. Demographic Characteristics, Descriptive Statistics and Correlations

To understand the demographic characteristics of the respondents, descriptive statistics and frequency analyses were conducted. Table 1 provides the frequencies, percentages, means, and standard deviations among the respondents. Among the 130 respondents, assistant managers provided the highest response rate at 40.0% (*n* = 44), followed by staff at 33.8% (*n* = 44), general managers at 21.5% (*n* = 28), and executive managers at 4.6% (*n* = 6). The results provide more information about execution in the field than in the planning aspects of digitalization considered by executives. In terms of a respondents’ working area, research and development departments showed the highest response rate with 44 respondents (33.8%), followed by strategy planning with 36 respondents (27.7%), manufacturing and production management with 26 respondents (20.0%), and quality control with 24 respondents (18.5%).

Table 2 shows mean and standard deviation for each variable as well as its critical ratio (CR), alpha (α) values, average variance extracted (AVE) and correlation coefficients. The AVE value of the construct was higher than the suggested minimum of 0.5. Cronbach’s alpha coefficient was used in a reliability analysis to confirm internal consistency; if the reliability of the measurement item was 0.7 or higher, the reliability was considered high. As shown in Table 2, the Cronbach’s alpha of the measurement item of this study was 0.747~0.890, which verified the internal consistency of the measurement item.

### 4.2. Validity and Reliability Analysis

The pre-test, which was conducted using the initial variables, confirmed the validity of the factors, except for a single factor in each dimension of social capital. Based on the results of the pre-test, factor and reliability analyses were conducted using SPSS 22.0 and AMOS 22.0 to determine the unity, reliability, and feasibility of healthcare digitalization, social capital, and performance. Six factors were extracted (see Table 3). One question was removed from each of the three dimensions of social capital, and all other research variables were factored out in the same way as the theoretical structure of previous research. The factor-loading value of the measurement items was 0.637~0.900 (all of which exceeded 0.6), and 74.138% of the total variance in data was explained.

### 4.3. Research Hypotheses Tests

**Figure 2:**
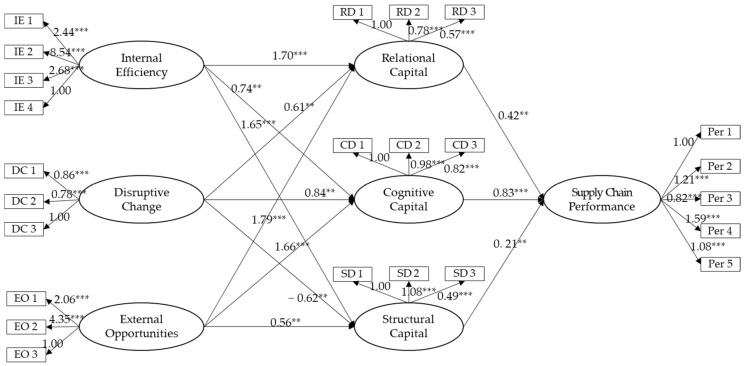
The research model results; ** *p* < 0.05, *** *p* < 0.01.

The AMOS 22.0 module was applied to analyze the relationships between healthcare digitalization and social capital (H1), and between social capital and supply chain performance (H2). The analysis results are shown in Figure 2. According to the model fit of the research model, the χ^2^ value was 945.802 (*p* = 0.000), which was not significant. However, χ^2^/df was 3.54, which was close to 3, which satisfies the model fit. The comparative fix index, incremental fit index and normed fit index were 0.94, 0.84, 0.93, respectively. The absolute fit indices of root mean square error of approximation and root mean square residual were 0.08 and 0.08. respectively. These results indicate a good fit between the model and the data.

As a result of the relationship between healthcare digitalization and social capital (H1), the effect of internal efficiency on relational capital was β = 1.70 (*p* = 0.002), on cognitive capital β = 0.74 (*p* = 0.029), and on structural capital β = 1.65 (*p* = 0.004). Next, the effect of disruptive change on relational capital was β = 0.61 (*p* = 0.047), on cognitive capital β = 0.84 (*p* = 0.046) and on structural capital β = −0.62 (*p* = 0.019). The effect of external opportunities on relational capital was β = 1.79 (*p* = 0.000), on cognitive capital β = 1.66 (*p* = 0.000), and on structural capital β = 0.56 (*p* = 0.029), validating H1.

According to the results of the relationship between social capital and supply chain performance (H2), the effect of relational capital on supply chain performance was β = 0.42 (*p* = 0.039). Next, the effect of cognitive capital on supply chain performance was β = 0.83 (*p* = 0.008). The effect of structural capital on supply chain performance was β = 0.21 (*p* = 0.046). H2 was therefore supported.

**Figure 3:**
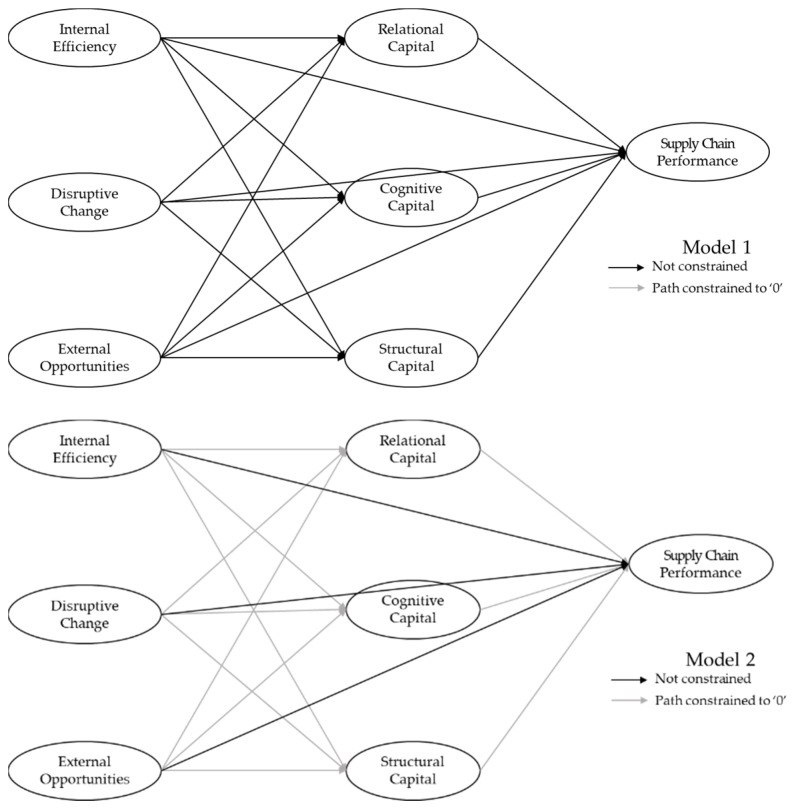
Alternative models to verify the mediating effect (Model 1 and Model 2).

The mediating effect of social capital on the relationship between healthcare digitalization and supply chain performance was also tested. To verify the effect, two alternative models (Model 1 and Model 2) were set and a χ^2^ difference test was performed. We verified the mediating effect by comparing the superiority between alternative models in the nested relation. To establish the nested relation, alternative models were based on the same latent and measured variables as the research model. The first alternative was Model 1, which set all the paths in the research model and additional paths (direct relationship between healthcare digitalization and supply chain performance). The second alternative was Model 2, which showed only a direct relationship between healthcare digitalization and supply chain performance. Model 2 was nested within Model 1, as shown in Figure 3.

To verify the mediating effect, the χ^2^ value was checked to determine the superiority of the alternative models. Because Model 1 and Model 2 were nested, they can be directly compared through a χ^2^ difference test. Model 1 had a lower χ2 value and fewer degrees of freedom than that of Model 2 (∆χ^2^ = 129.494, ∆*df* = 12). In other words, Model 1 produced a decrease of 12 degrees of freedom relative to Model 2, but because the χ^2^ value decreased sufficiently to offset the decrease in degrees of freedom, it can be said that Model 1 was superior to Model 2.

According to the path coefficient value of Model 1 used to support the superiority of Model 1, the healthcare digitalization and social capital path coefficients that were not included in Model 2 were significant. The effect of internal efficiency on relational capital was β = 1.66 (*p* = 0.001), on cognitive capital β = 0.69 (*p* = 0.033), and on structural capital β = 1.59 (*p* = 0.004). Next, the effect of disruptive change on relational capital was β = 0.65 (*p* = 0.045), on cognitive capital β = 0.57 (*p* = 0.026), and on structural capital β = −0.47 (*p* = 0.017). The effect of external opportunities on relational capital was β = 1.74 (*p* = 0.000), on cognitive capital β = 1.61 (*p* = 0.000) and on structural capital β = 0.57 (*p* = 0.026).

The social capital and supply chain performance path coefficients not included in Model 2 were also significant. At first, the effect of relational capital on supply chain performance was β = 0.42 (*p* = 0.037). Next, the effect of cognitive capital on supply chain performance was found to be β = 0.62 (*p* = 0.027). The effect of structural capital on supply chain performance was found to be β = 0.14 (*p* = 0.043). We therefore concluded that the suitability of Model 1, which includes these significant paths, had increased. In addition, the direct effect of digitalization on supply chain performance was small and a relatively large part was mediated by social capital, supporting H3.

## 5. Discussion

Digitalization of a supply chain in healthcare industry improves access to market demand and customer information. Healthcare manufacturers can, therefore, develop seamless partnerships with strong interrelationships. In addition, the continuity of relationships with existing suppliers can be strengthened, and opportunities to establish relationships with new suppliers increased. Through healthcare digitalization, social capital, a potential resource and asset that accumulates in supply chain flows, has a positive effect. A detailed discussion of the results of the analysis reveals three critical findings.

First, healthcare digitalization has a positive effect on the formation of social capital. In healthcare manufacturing, strategic relationships are crucial, as supply chain networks and production processes influence the intended use of output. Digitalization increases the visibility of supply chain networks, enables planning in strategic relationships, and uncovers opportunities to establish new relationships. In addition, the range of products can be expanded and the complexity of the design process addressed using digital products and services [8,51]. Healthcare digitalization has been found to have a positive impact on social capital formation, accelerating and promoting the formation and sharing of social capital. Disruptive changes in healthcare digitalization were found to negatively affect structural capital. Social capital is formed by strong social networks among partnership firms in a supply chain, and it is possible to establish mutually cooperative relationships and secure supply chain visibility. However, we confirmed that digital manufacturing technology platforms can prove disruptive of the platform is the focus, rather than mutually cooperative relationship-building [20,52].

Second, we found that relational, cognitive, and structural capital (as a lower dimension of social capital) had positive effects on supply chain performance. For healthcare manufacturing companies, it is essential to secure timely and accurate information to manage the flow of products and services. This can be realized by establishing close and interactive relationships among partnership firms. We confirmed that the formation of social capital in a supply chain enables visualization of the entire supply chain process and has a positive effect on real-time information-sharing between key elements. For SCM to be successful, mutually cooperative relationships between partners in a supply chain are essential, and a trusting relationship for information-sharing must be established. In addition, sharing a common vision and goals within a supply chain plays a key role in ensuring efficient operations by promoting relational accessibility and collaborative commitments among partnership firms [11,14,53]. We confirmed that the formation of social capital of small and medium-size enterprises in a supply chain can have a positive effect on agile SCM by enabling the efficient operation and resource management of companies.

Finally, we found that social capital plays a mediating role in the relationship between healthcare digitalization and supply chain performance. Healthcare manufacturers must be flexible when responding to changes in non-standard orders, specific order requirements, immediate demands, and trends through active internal and external communications. Access to product-specification demand and market-demand information can be improved by digitalization of the supply chain, and shortening delivery cycle times. Close interactions among partnership firms are beneficial, and shared business values should be embraced. To form a supply chain network based on a digital manufacturing technology, a cross-functional team with partnership firms must be formed and a team for organizational development must be established [21,54]. The direct effect of healthcare digitalization on supply chain performance is small, and relatively large parts are mediated and influenced by social capital.

## 6. Conclusions

With the spread of COVID-19, daily lives have changed dramatically and the industrial ecosystems are undergoing major paradigm shifts. As a result, manufacturing sectors needs to strengthen value chains where its businesses are located. Digitalization based on digital manufacturing technologies such as automation, AI and IoT is emerging as a major opportunity for manufacturers. The prolonged COVID-19 pandemic means manufacturing costs are continuing to increase while production workforces are shrinking, and digitalization is expected to increase the productivity of the manufacturing industry. We conducted a study of the effects of digitalization that can be expected in global supply chains due to the globalization of existing production bases. Based on the research objectives and results, the following implications are likely.

Until now, digitalization research related to supply chains has been conducted primarily through qualitative research. Although the importance and necessity of digitalization has been enhanced through such studies, empirical evidence on the performance of manufacturing companies that have applied digitalization is lacking. Supply chains of healthcare manufacturers are highly vulnerable to the effects of changing technological trends, and digitalization can have a profound effect on performance. In addition, as the formation of social capital in a supply chain network accelerates, the efficient delivery of product specifications and market-trend information of value to healthcare manufacturers is essential. Our findings show that the performance of healthcare manufacturer can be improved by improving supply chain visibility with digitalization and the rapid creation of social capital.

The advent of digital manufacturing technologies requires that manufacturer take risks involving innovation and choose strategic alternatives for competitive advantage. The existing manufacturing industry environment caused by globalization can adapt to the challenges presented by COVID-19. Supply chain managers need a clear understanding of digitalization to apply the appropriate AI and automation tools. They must also maintain supply chain partnerships using social capital that helps achieve target performance. The evaluation of existing partnerships and the discovery of new partnerships can be accomplished by creating social capital within a supply chain network. Social capital formed in a supply chain should be considered in future research to confirm the performance aspects of digitalization.

Since this study attempted to verify the impact of digitalization on healthcare manufacturing companies, it is difficult to generalize the entire healthcare industry based on the results of the study. Therefore, through future research, we intend to proceed with research on the impact of digitalization in the new normal era caused by COVID-19. In addition, by combining digitalization and servitization, it intends to derive implications for the healthcare service industry.

## Figures and Tables

**Table 1:** Demographic characteristics for respondents (*n* = 130).

Variable	N	%	Mean	SD
Position	130		1.97	0.86
Executive manager	6	4.6		
General manager	28	21.5		
Assistant manager	52	40.0		
Staff	44	33.8		
Department	130		2.74	1.15
Manufacturing/production	26	20.0		
R&D	44	33.8		
Quality control	24	18.5		
Strategy planning	36	27.7		

Note: SD = standard deviation.

**Table 2:** Descriptive statistic, CR, Alpha, AVE and correlation analysis for constructs.

Variables	Mean	SD	CR	α	Pearson Correlations (*n* = 130)						
					1	2	3	4	5	6	7
1. IE	3.83	0.519	0.853	0.827	(0.758)						
2. DC	4.23	0.488	0.816	0.765	0.661 *	(0.587)					
3. EO	3.91	0.516	0.865	0.753	0.673 *	0.497 **	(0.668)				
4. RD	3.92	0.494	0.886	0.803	0.513 **	0.530 *	0.305 **	(0.754)			
5. CD	3.92	0.642	0.835	0.769	-0.277 **	0.150 **	0.333 **	0.205 **	(0.677)		
6. SD	3.71	0.574	0.834	0.747	0.391 **	0.211 *	0.344 *	0.334 **	0.487 **	(0.728)	
7. PE	4.10	0.730	0.900	0.890	0.219 *	0.270 **	0.169 *	0.280 *	0.191 *	0.207 *	(0.676)

* *p* < 0.05, ** *p* < 0.01, Note: Parenthesis indicates AVE values of the individual constructs. Off-diagonal scores are the squared correlations between the constructs. IE = internal efficiency, DC = disruptive change, EO = external opportunities, RD = relational capital, CD = cognitive capital, SD = structural capital, PE = performance.

**Table 3:** Factor analysis for independent, mediated and dependent variables.

Factors	1	2	3	4	5	6	7	Eigen Value
Internal efficiency								4.474
IE 1	0.847							
IE 2	0.651							
IE 3	0.760							
IE 4	0.824							
Disruptive change								4.205
DC 1		0.784						
DC 2		0.637						
DC 3		0.775						
External opportunities								3.321
EO 1			0.813					
EO 2			0.676					
EO 3			0.836					
Relational capital								2.466
RD 1				0.900				
RD 2				0.754				
RD 3				0.762				
Cognitive capital								2.211
CD 1					0.773			
CD 2					0.842			
CD 3					0.773			
Structural capital								1.811
SD 1						0.848		
SD 2						0.775		
SD 3						0.646		
Performance								1.512
PE 1							0.845	
PE 2							0.840	
PE 3							0.738	
PE 4							0.834	
PE 5							0.844	

Note: Factor loadings below 0.50 were removed from the table, IE = internal efficiency, DC = disruptive change, EO = external opportunities, RD = relational capital, CD = cognitive capital, SD = structural capital, PE = performance.

## Data Availability

Data are not publicly available due to data generated by the survey form developed for this study. Simplified survey form is presented in Appendix A.

## Appendix A

Internal efficiency
  - IE1 Facilitates access to information related to demand markets and customers.
  - IE2 Facilitates strategic planning of the supply chain.
  - IE3 Brings about cost savings.
  - IE4 Makes easily to develop employee competencies and job-related skills.
Disruptive change
  - DC1 Maintains cooperation with existing supply chain partners through digitalization.
  - DC2 Facilitates forming partnerships with new supply chain partners through digitalization.
  - DC3 Increases supply chain flexibility through digitalization.
External opportunities
  - EO1 Facilitates communication within the company through digitalization.
  - EO2 Facilitates communication with external stakeholders through digitalization.
  - EO3 Facilitates market activities related to demand and supply market demand through digitalization.
Relational capital
  - RD1 Close interaction with partner companies from various angles.
  - RD2 Forms a partnership relationship based on mutual trust, respect, friendship.
  - RD3 Establishes a mutual win-win strategy based on high reciprocity.
Cognitive capital
  - CD1 Strives to increase mutual benefits, profits with partners.
  - CD2 Shares common business values, goals with partners.
  - CD3 Shares the strategic direction, vision for the future with partners.
Structural capital
  - SC1 Jointly participates in social activities with partners.
  - SC2 Forms multifunctional teams with partners.
  - SC3 A co-location method is in progress with a partner.
Supply chain performance
  - PE1 Processes non-standard orders in the supply chain.
  - PE2 Launches new products rapidly.
  - PE3 Quickly adjusts the degree of production increase or decrease in an immediate response to changes in consumer demand.
  - PE4 Crossover activities beyond the boundaries of trade partners in supply chain.
  - PE5 Reduces the order-delivery cycle time.
